# A Simple Three-Dimensional In Vitro Culture Mimicking the In Vivo-Like Cell Behavior of Bladder Patient-Derived Xenograft Models

**DOI:** 10.3390/cancers12051304

**Published:** 2020-05-21

**Authors:** Robson Amaral, Maike Zimmermann, Ai-Hong Ma, Hongyong Zhang, Kamilla Swiech, Chong-Xian Pan

**Affiliations:** 1 Department of Pharmaceutical Sciences, School of Pharmaceutical Sciences of Ribeirao Preto, University of Sao Paulo, 14040-903 Sao Paulo, Brazil; amaral.r@usp.br; 2 Department of Internal Medicine, Division of Hematology and Oncology, University of California Davis Medical School, Sacramento, CA 95817, USA; maikezimmermann@gmx.net (M.Z.); hyzh@ucdavis.edu (H.Z.); cxpan9@yahoo.com (C.-X.P.); 3 Department of Urology, UC Davis Comprehensive Cancer Center, University of California Davis Medical School, Sacramento, CA 95817, USA; ahmma@ucdavis.edu; 4 VA Northern California Health Care system, Mather, CA 95655, USA; 5 (Current address): Department of Faculty of Medicine, Harvard Medical School, West Roxbury, MA 02115, USA

**Keywords:** 3D cell culture, PDX models, tumor spheroids, bladder cells, ultra-low attachment plates, Matrigel

## Abstract

Patient-derived xenograft (PDX) models allow for personalized drug selection and the identification of drug resistance mechanisms in cancer cells. However, PDX models present technical disadvantages, such as long engraftment time, low success rate, and high maintenance cost. On the other hand, tumor spheroids are emerging as an in vitro alternative model that can maintain the phenotype of cancer cells long enough to perform all assays and predict a patient’s outcome. The present work aimed to describe a simple, reproducible, and low-cost 3D in vitro culture method to generate bladder tumor spheroids using human cells from PDX mice. Cancer cells from PDX BL0293 and BL0808 models, previously established from advanced bladder cancer, were cultured in 96-well round-bottom ultra-low attachment (ULA) plates with 5% Matrigel and generated regular and round-shaped spheroids (roundness > 0.8) with a diameter larger than 400 μm and a hypoxic core (a feature related to drug resistance in solid tumors). The responses of the tumor spheroids to the antineoplastic drugs cisplatin, gemcitabine, and their combination were similar to tumor responses in in vivo studies with PDX BL0293 and BL0808 mice. Therefore, the in vitro 3D model using PDX tumor spheroids appears as a valuable tool that may predict the outcome of in vivo drug-screening assays and represents a low-cost strategy for such purpose.

## 1. Introduction

Bladder cancer is considered a serious public health problem due to its high recurrence rate and mortality, with approximately 150,000 deaths in the world per year [1,2]; thus the development of new therapeutic approaches is of paramount importance. The current approach of ‘one treatment fits all’ is not considered the best strategy since it does not take into account the patient-specific genetic variability that has a potential influence on the response to drugs [3]. One of the approaches of personalized oncology is based on the prediction of the response of a patient’s tumor to anti-neoplastic drugs to help the oncologist plan the best treatment for that patient [4]. Patient-derived xenografts (PDX) in immune-compromised mice have emerged as an in vivo model developed for drug screening [5]. PDX models allow for the expansion of a tumor fragment, maintaining the intratumor heterogeneity, histopathological features, metastatic behavior, and gene expression profile of the original tumor [6]. We previously showed that PDXs retained 92%–97% of the genetic alterations present in the parental patient cancers [7]. This model facilitates personalized drug selection and the identification of tumor resistance mechanisms [8]. However, PDX-based models also present some technical disadvantages such as low success rate (30%–40%), long engraftment time (2 to 8 months), high cost, limited statistical power, and low potential for application to high-throughput studies [9].

To overcome these limitations, three-dimensional (3D) cultures using patient-derived cells have been suggested as an alternative in vitro model [10,11,12,13,14]. This method generates tumor spheroids that have the objective of maintaining the phenotype of the original tumor cells long enough to perform all assays and predict the patient outcome [15,16]. In contrast to traditional bi-dimensional (2D) cultures, 3D cultures are able to recapitulate many aspects of the native tumor microenvironment, such as cell–cell and cell–extracellular matrix (ECM) interactions [17]. The poor diffusion of oxygen and nutrients throughout a spheroid results in the presence of proliferative cells in the outside layer and the generation of a hypoxic region with quiescent cells in the innermost layers [18]. Hypoxia affects the expression of genes that are related to anti-cancer drug resistance, which can be observed in solid tumors [19] and is impossible to reproduce in 2D cultures.

When primary cancer cells are subjected to 3D conditions, the heterogeneous composition of the original tumor can be largely conserved [20], and the tumor spheroids can recapitulate the morphological and genetic features of the parent tumor [21]. From this perspective, PDX models can be a source of human cancer cells to generate tumor spheroids (PDX tumor spheroids), overcoming the issue of limited collection of multiple patient samples. PDX mice can be used for in vivo serial propagation of tumor tissue, providing sufficient material for repeated experiments [22]. Herein, we describe the development of a simple, reproducible, and low-cost 3D in vitro culture method for PDX bladder tumor cells that mimic the in vivo cell behavior and response to therapeutics.

## 2. Results and Discussion

### 2.1. Characterization of PDX Tumor Spheroids

**Figure 1:**
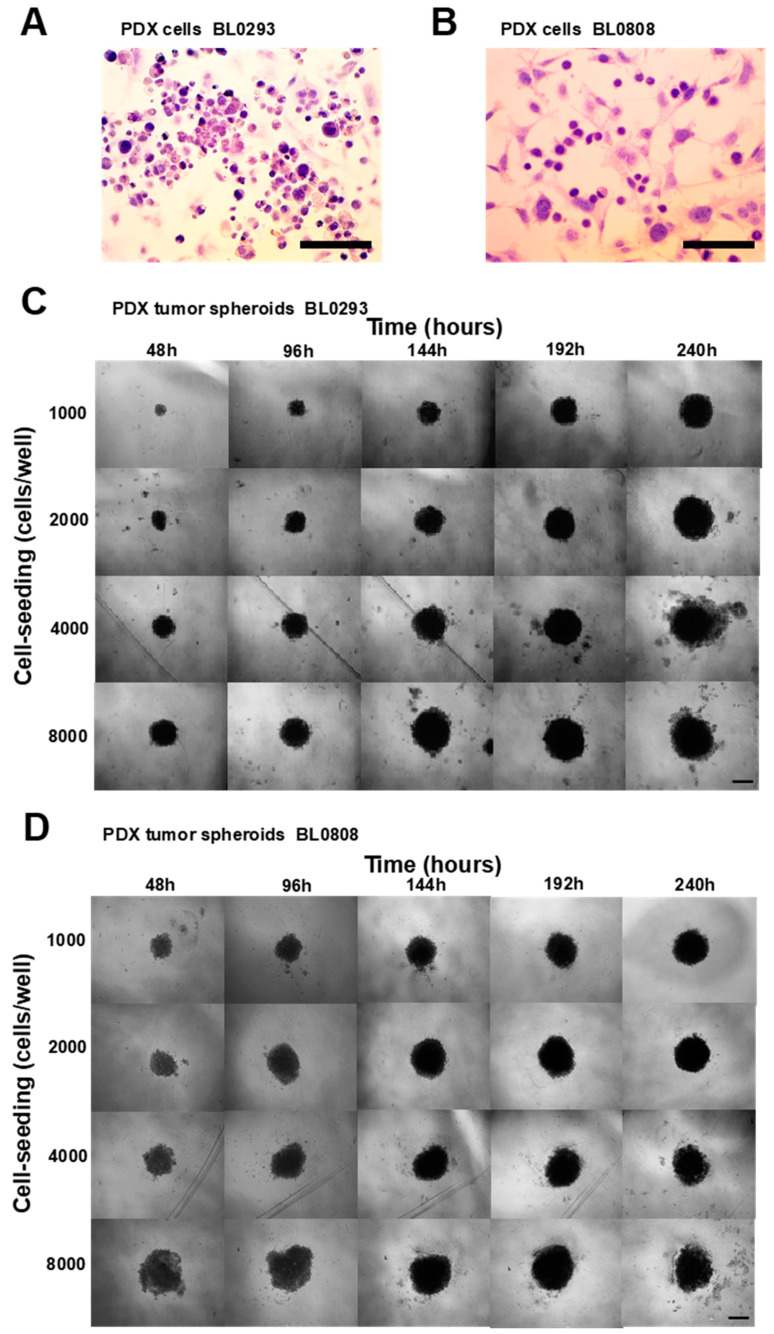
Bright-field microscopic images of patient-derived xenograft (PDX) tumor cells BL0293 (**A**) e BL0808 (**B**) stained with hematoxylin and eosin (tumor cells purplish/bluish-colored and fibroblasts pinkish-colored); 40× magnification, scale bar = 100 μm. Phase-contrast microscopic images of PDX tumor spheroids BL0293 (**C**) and BL0808 (**D**) at different cell-seeding concentrations, cultured for 10 days; 10× magnification, scale bar = 500 μm.

The culture of single cells isolated from tumor fragments presented a predominance of tumor cells (purplish/bluish) with respect to fibroblasts (pinkish), as seen in slides stained with a hematoxylin and eosin Y (H&E) solution (Figure 1A,B). The results showed the efficacy of the method in isolating large amounts of tumor cells from the stroma (partial digestion followed by straining and centrifugation cycles at different speeds and times). The method described was based on protocols retrieved from the literature for other types of tumors [23,24,25]. Using the forced floating method in round-bottom 96-well ultra-low attachment (ULA) plates and RPMI culture medium supplemented with 5% Matrigel, PDX tumor spheroids BL0293 and BL0808 were formed within 48 h at all cell seeding densities used (Figure 1C,D). Due to the concave form of the wells, only one spheroid was generated per well. In the first hours of culture, cellular aggregates formed mainly by interactions between cadherin proteins present in the cell membranes [26,27]. The secretion of extracellular matrix and the interaction of these proteins with the cytoskeleton guarantees the rigidity of the cell aggregates and consequent compaction of the spheroids [28].

**Figure 2:**
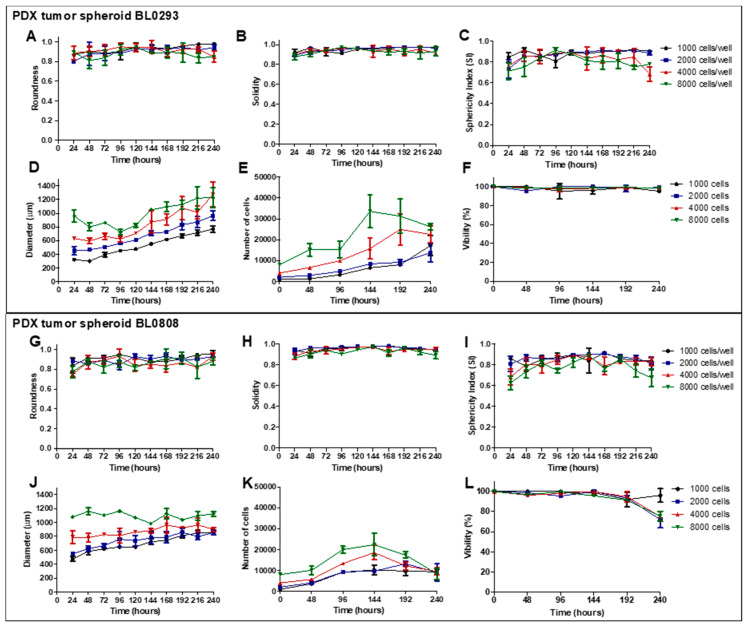
Characterization of PDX tumor spheroids BL0293 (**A**–**F**) and BL0808 (**G**–**L**) based on the evaluation of shape parameters (roundness, solidity, and sphericity index (SI)), size (Feret’s diameter), cell growth, and viability for 240 h of culture, starting from different cell-seeding densities. Data displayed as mean ± SD referring to one experiment with three technical replicates (wells) per condition (*n* = 3).

The analysis of spheroids’ shape parameters is an important feature in the choice of the culture conditions (cell-seeding density and time of culture), since regular and well-rounded spheroids are more stable when used for in vitro assays and present less variability when used in drug screening [29,30,31]. The roundness values were higher than 0.8 for both PDX tumor spheroids during 240 h of culture (Figure 2A,G), indicating a high circularity that ensures the total compactness of the cell aggregates [32]. The solidity values, close to 0.90 in both models (Figure 2B,H), indicated the presence of a regular surface [33]. Another shape parameter analyzed was the sphericity index (SI), which varied less in cultures seeded at low cell concentrations for both models (Figure 2C,I). Tumor spheroids generated with 1000 and 2000 cells/well maintained ab SI over 0.85 most of the culture time, indicating the maintenance of the spherical shape during the assay. SI values lower than 0.80 were observed for spheroids generated with 4000 and 8000 cells/well. Generally, all PDX tumor spheroids presented a regular and round morphology according to the parameters analyzed.

The mean diameter of PDX tumor spheroids BL0293 seeded with 1000, 2000, 4000, and 8000 cells/well ranged from 321 (± 20) to 770 (± 44), 451 (± 64) to 966 (± 74), 631 (± 17.51) to 1274 (± 183.73), and 960 (± 154) to 1225 (± 250) μm, respectively (Figure 2D). The greatest variation in size was observed in cultures seeded with 1000 cell/well (2.4-fold increase) throughout the 10 days of culture. On the other hand, the cultures with the highest cell density showed only a 1.27-fold increase in size. These results are in agreement with those from the spheroid cell growth analysis, since the greatest increase in cell number, 17.2-fold (± 4.7), was also observed in cultures seeded with 1000 cell/well. Spheroids in other conditions showed 6.8- (± 2.2), 6.0- (0.92), and 4.76-fold (± 0.67) increases in cell number, respectively (Figure 2E). The cells in the spheroids maintained a viability higher than 90% at all seeding densities used (Figure 2F). However, a drop in the number of cells was observed in spheroids seeded with 4000 and 8000 cell/wells after 192 and 144 h of culture, respectively. The culture of PDX BL0808 cells formed larger spheroids in the early stages of culture (probably due to the lower degree of cell compaction); however, the variation in size was smaller than that observed for PDX spheroids BL0293 (less than twofold increase). When seeding of 1000, 2000, 4000, and 8000 cells/well, the spheroids mean diameter ranged from 477 (± 30) to 846 (± 22), 548 (± 12) to 855 (± 36), 788 (± 90) to 902 (± 31), and 1077 (± 33) to 1124 (± 58) μm (Figure 2J), respectively. The number of cells increased 11.5 (± 1.32), 6.66 (± 0.63), 4.62 (± 0.87), and 3 (± 0.36) times, respectively, throughout the 10 days of culture (Figure 2K). A drop in the cell number was also observed in spheroids seeded with 4000 and 8000 cells/wells after 144 h. Up to 144 h of culture, cell viability in all culture conditions was above 90% (Figure 2L). After that point, the cell viability of the spheroids seeded with 2000, 4000, and 8000 cells/well dropped to 72.00% (± 8.18%), 75.00% (± 5.00%), and 75.33% (± 5.03%), respectively.

The generation of tumor spheroids with uniform geometry and homogeneous sizes is some of the most relevant factors for choosing 3D culture conditions, since these properties allow uniform diffusion of oxygen and nutrients within the tumor spheroids, influencing the internal organization of the cells [4]. Tumor spheroids with different profiles will result in different responses, increasing the variability of the results [31]. In addition, 3D tumor spheroids have the advantage of inducing common cellular features associated with drug resistance, such as cellular senescence, hypoxia, and stem-like properties [34,35]. These features are closely dependent on tumor spheroid size, which can be controlled by the initial cell seeding concentration and culture time. Tumor spheroids can also recreate the pathophysiological oxygen and nutrient gradients that are found in the original tissue [36]. Cells in the outer layer of a tumor spheroid receive a higher supply of oxygen and nutrients, showing a proliferative behavior. The presence of proliferative cells in PDX bladder tumor spheroids was evidenced by the increase in tumor spheroid size and cell number throughout culture. An intermediate layer is composed of quiescent cells, and an inner hypoxic core, resulting from the limited diffusion of oxygen and nutrients, is also observed in in vitro tumor spheroids. Therefore, tumor spheroids must be large enough to allow the formation of this chemical gradient and small enough not to cause aberrations like a secondary necrotic core that may affect the accuracy of the drug efficacy test [37]. Considering this, the suitable tumor spheroid size for in vitro tumor cell-based assays ranges between 300 and 500 μm [36,38,39,40].

**Figure 3:**
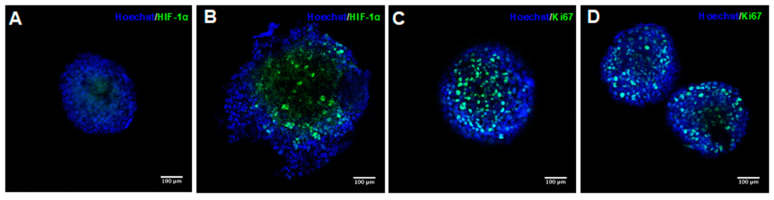
Fluorescence confocal microscopy images of PDX tumor spheroids BL0293 (**A**,**C**) and BL0808 (**B**,**D**). Cell nuclei stained in blue, hypoxic inducible factor 1α (HIF-1α) and Ki67 protein stained in green; 20× magnification, scale bar = 100 μm.

According to the values of the shape parameters and diameters, six-day PDX tumor spheroids formed with 1000 cells/well were selected to perform a drug sensitive assay. In order to ascertain whether such spheroids mimic some of the properties observed in solid tumors, such as the presence of proliferating cells in the outer layer and hypoxic cells in the inner region of the spheroids, the spheroids were submitted to immunofluorescent staining. It was possible to observe a small region of hypoxia in the inner region of the tumor spheroids BL293 (Figure 3A). This region was more visible in BL0808 spheroids and seemed not to be restricted to the core of the spheroids (Figure 3B). This could result from a compromised integrity of the spheroids’ 3D structure due to fixation, permeabilization, and various washing cycles during the staining process. The irregular edges observed in the spheroids BL0808 support this inference. The same staining profile was observed for spheroids derived from the bladder cancer cell lines RT4 and T24 [41]. Low rates of oxygen diffusion into a solid tumor in vivo induce the expression of the hypoxic inducible factor 1α (HIF-1α) that target genes such as the vascular endothelial growth factor A (VEGF-A), the glucose transporter 1 (GLUT1), and the carbonic anhydrase 9 (CA-IX), promoting angiogenesis [42]. The expression of HIF target genes was also observed in tumor spheroids and has been associated with cell survival through the regulation of metabolic reprograming and repression of pro-apoptotic signaling and proliferation [43]. Both types of spheroid showed the presence of proliferative cells marked with an anti-Ki67 antibody (Figure 3C,D). By analyzing the images, it was possible to verify that the proliferative cells seemed to be spread across the spheroids.

### 2.2. Drugs Response in PDX and PDX Tumor Spheroid Models

**Figure 4:**
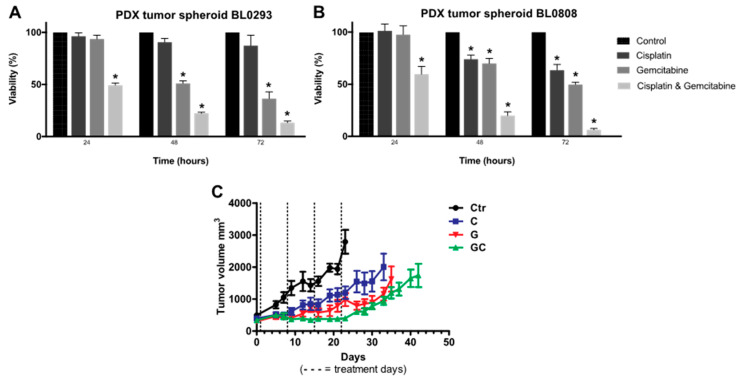
In Vitro drug response. Cell viability of six-day PDX tumor spheroids BL0293 (**A**) and BL0808 (**B**) treated with 10 μM cisplatin, gemcitabine, and combination thereof for 24, 48, and 72 h. Data displayed as cell viability mean ± SD, referring to two experimental replicates with three technical replicates each (*n* = 6). T-student statistical method with * *p* < 0.01 vs. control. In Vivo drug response: variation of tumor volume for PDX mice BL0808 (**C**). Mice were randomly assigned to the treatment groups and treated with cisplatin at 2 mg/kg (intravenous injection every seven days at 3 doses), gemcitabine at 150 mg/kg (intraperitoneal injection every seven days at 4 doses), or their combination. Data displayed as tumor volume mean ± SEM of a group with six animals (*n* = 6).

Six-day PDX tumor spheroids seeded with 1000 cells/well were treated with the antineoplastic drugs cisplatin, gemcitabine, and a combination thereof, at a concentration of 10 μM. The cell viability of PDX tumor spheroids BL0293 decreased by 50% after 24 h of treatment with the combination of cisplatin and gemcitabine (Figure 4A). After 48 and 72 h, the viability reached 22.3% (± 1.7%) and 13.3% (± 2.8%), respectively. A sensitive response was also observed with gemcitabine treatment, which decreased the cell viability to 36.5% (± 11.1%) after 72 h of exposure. No statistical difference was observed between control and tumor spheroids treated with cisplatin. With these results, we could conclude that PDX tumor spheroids BL0293 were highly sensitive to gemcitabine and the combination of cisplatin and gemcitabine and were resistant to cisplatin. A similar behavior was observed in PDX BL0293 mice according to the in vivo data obtained in a previous work [7]. In the first days of treatment, before the possible development of resistance, the tumor volume in PDX BL0293 mice decreased upon treatment with gemcitabine and the cisplatin and gemcitabine combination. PDXs BL0293 were generated using an advanced bladder cancer sample from a patient that had not been treated and were also more sensitive to the combination of drugs rather than the individual ones. The initial resistance of PDX tumor spheroids to cisplatin, a DNA-damaging agent, can be associated with the overexpression of nucleotide excision repair (NER) genes, commonly observed in ovarian, glioma, bladder, and lung cancer cells [44]. Gemcitabine incorporates into DNA and decreases the expression of two key proteins of the NER system: ERCC1 and XPA [45]. Thus, when used in combination, the inhibition of the NER system by gemcitabine increases the formation of platinum adducts in the DNA by cisplatin [46]. The combination of cisplatin and gemcitabine is commonly used as first-line chemotherapy for advanced bladder cancer and displays synergistic antitumor activity [47]. This synergistic anti-tumor activity was also reported in spheroids formed by neuroblastoma cells [48].

The combination of cisplatin and gemcitabine was also more effective for PDX tumor spheroids BL0808, showing cell viability of 59.5% (± 13.2%), 20.0% (± 6.2%) and 6.8% (± 1.4%) after 24, 48, and 72 h of exposure, respectively (Figure 4B). In the first 24 h of treatment, no statistical difference was observed between control tumor spheroids and spheroids treated with cisplatin or gemcitabine treatment. The viability of the tumor spheroids treated with cisplatin decreased to 74.0% (± 7.2%) and 63.5% (± 9.6%) after 48 and 72 h, showing a partial resistance of PDX tumor spheroids BL0808. In contrast to BL0293 spheroids, BL0808 tumor spheroids were less sensitive to gemcitabine, showing a cell viability of 70.0% (± 8.4%) and 49.7% (± 4.0%). These results are also similar to the overall results obtained with PDX BL0808 (Figure 4C). PDX BL0808 was established from an advanced bladder cancer specimen of a patient who was previously treated with gemcitabine and cisplatin. Even though there were no differences in tumor volume in the first days of treatment, the combination of cisplatin and gemcitabine was more effective in decreasing the volume of the tumor compared to the other two treatments. Indeed, several works in the literature show that PDX and PDX-derived tumors obtained from previously treated patients tend to be more drug-resistant than those from untreated patients [49].

Personalized oncology treatments have emerged in recent years due to improvements in molecular characterization and pharmacogenetic profiling techniques [50]; however, several studies have shown that in many cases, the detection of tumor mutations does not necessarily confirm the efficacy of a therapy [51]. For example, in a study by Pauli and collaborators, only 0.4% of somatic changes in cancer cells are targets for U.S Food and Drug Administration (FDA)-approved chemotherapy [20]. Functional assays on PDX tumor cells in 3D culture models are emerging as a possible strategy to provide a more precise characterization of drug efficacy than that offered by any omics-only-based approach [52]. Likewise, recent studies have reported a positive correlation in co-clinical trials between a patient’s response to treatment and results obtained in preclinical trials using patient-derived cells cultured in 3D models [53,54]. A preliminary drug screening using 3D in vitro models with PDX tumor cells may eliminate those drugs that will probably be ineffective in animal models. This approach may result in a reduction of cost and time of drug screening processes.

## 3. Materials and Methods

### 3.1. Bladder Cancer PDX Models

Two PDXs of bladder cancer were used in this work, BL0293 and BL0808. All experiments were performed in compliance with institutional guidelines and approved by the Animal Use and Care Administrative Advisory Committee at the University of California, Davis (Sacramento, CA, USA) (UC Davis Institutional Care and Use Committee IACUC 19564). PDX BL0293 was previously developed with fresh sample from an advanced bladder cancer (myoinvasive, high grade) from a patient with no prior chemotherapy treatment, according to Pan et al., 2015. PDX BL0808 was obtained from The Jackson Laboratory Cancer Center (JAXCC, Sacramento, CA, USA) from an advanced bladder cancer of a patient previously treated (5 months before sample collection) with neoadjuvant chemotherapy (4 cycles gemcitabine/cisplatin) (JAXCC reference J000101121). Both PDXs were created using female NOD scid gamma severe combined immunodeficient (NSG) mice (5–8 weeks of age, body weight: 20–25 g) by subcutaneous injection in the flank of approximately 1 mm^3^ of cancerous tissue from a patient’s primary tumor.

### 3.2. Isolation and Culture of Bladder Tumor Cells from PDXs

Bladder tumors from PDXs BL0293 and BL0808 were mechanically fragmented (scalpel blade) and partially digested with an enzyme solution of 1% (*v/v*) collagenase type 1 (Gibco™, Thermo Fisher Scientific, Waltham, MA, USA) and 5% (*v/v*) dispase (Thermo Fisher Scientific, Waltham, MA, USA) at 37 °C in a water bath for 40 min to 1 h, shaking each 10 min. The partial digestion step guaranteed the maintenance of tumor cell clusters in the suspension. After filtration, the tumor cell clusters were withheld in a sterile 100 μm-size nylon mesh (Milipore^®^, Merck KGaA, Darmstadt, Gemany). This was the first step to separate tumor cell clusters from certain stromal components. The tumor clusters were washed with PBS and centrifuged at 530 *g* for 5 min. The supernatant was then removed. The pellet was washed again with PBS and centrifuged at 530 *g* for 1 min. The fast centrifugation allowed the precipitation of tumor cell clusters that were heavier than single cells (most of them being fibroblasts) present in the supernatant. The supernatant was removed. The centrifugation cycles were repeated up to three times to ensure the removal of fibroblasts and other stromal cells as much as possible. Finally, the tumor cell clusters where totally dissociated into single cells by a 0.05% trypsin–ethylenediaminetetraacetic acid (EDTA) solution (Gibco) at 37 °C. The single tumor cells were cultured in 6-well plates with RPMI-1640 culture medium (Gibco) supplemented with 10% (*v/v*) fetal bovine serum (FBS, Thermo Fisher Scientific, Waltham, MA, USA), 1% (*v/v*) penicillin–streptomycin (Invitrogen™, Thermo Fisher Scientific, Waltham, MA, USA ), 1% (*v/v*) non-essential amino acids (Gibco), and 1% (*v/v*) L-glutamine (Gibco). The cells were stained with hematoxylin and eosin Y solutions (H&E). Bright-field images were obtained using a phase-contrast microscope with a live video microscope digital camera (AmScope, Irvine, CA, USA).

### 3.3. Generation and Characterization of PDX Tumor Spheroids

PDX cells were cultured in 96-well round-bottom ULA plates (Costar^®^, Corning, New York, NY, USA) at different cell seeding concentrations (1000, 2000, 4000, and 8000 cells/well) to generate spheroids by the forced floating technique, according to our previous work with the bladder cancer cell line RT4 [55]. Only one spheroid was obtained per well (total of 45 spheroids for each condition). The culture medium was supplemented with 5% of Matrigel (BD Biosciences, San Jose, CA, USA). The plates were centrifuged at 300 *g* for 10 min to improve cell aggregation. The medium was changed every two days by removing 50% of it from each well and adding 50% of fresh medium with 5% Matrigel.

The tumor spheroids formed were characterized in terms of their shape and size using the open-source software ImageJ (National Institutes of Health, Bethesda, MD, USA). The parameters analyzed by the software were roundness, solidity, circularity, and Feret’s diameter. The SI was calculated by the square root of the circularity parameter [56]. Three wells (3 spheroids) for each condition were analyzed per day. One bright-field image of the whole spheroids was taken from each well using a digital camera (AmScope) coupled to an inverted microscope (Olympus, Shinjuku, Japan) (10× objective). The images were automatically processed using macros from Image J, as described by Ivanov and collaborators [38]. The results of the shape parameters and size were plotted against time using the software for data analysis Prism version 7.0 (GraphPad Software Inc., San Diego, CA, USA) and displayed as mean ± SEM, referring to one experiment with three technical replicates (wells) per condition (*n* = 3). Cell growth and viability were analyzed by the Trypan blue dye exclusion test using a Neubauer chamber (Boeco, Hamburg, Germany), and the increase was evaluated by dividing the highest cell number reached by the initial number. Three wells (3 spheroids) for each condition (initial cell seeding) were analyzed every two days (from 24 to 240 h of culture). The results of cell number and viability were plotted against time using the software for data analysis Prism version 7.0 (GraphPad Software) and displayed as mean ± SEM, referring to one experiment with three technical replicates (wells) per condition (*n* = 3). The presence of hypoxic and proliferating cells was analyzed by an immunostaining technique. Six-day PDX tumor spheroids seeded with 1000 cell/well were fixed and permeabilized with 4% paraformaldehyde and 1% Triton X-100 in PBS, following a protocol described by Gheibi and collaborators [13]. The spheroids were stained with 1 μg/mL of Hoechst Stain 33342 (Cell Signaling Technology, Danvers, MA, USA), 1:250 anti-human hypoxic inducible factor (HIF) 1α (BD Transduction Laboratories^TM^), and 1:250 anti-human Ki67 8D5 (Cell Signaling Technology, Danvers, MA, USA). The spheroids were also treated with 1:200 goat anti-mouse IgG Antibody (H + L)–fluorescein isothiocyanate (FITC) conjugate (Millipore).

### 3.4. Drug Treatment of BL0808 PDX Mice and BL0808 and BL0293 PDX Tumor Spheroids

In Vivo drug treatment: the tumors (passages 2–4) in PDX BL0808 mice were allowed to grow to about 200 mm^3^ before being assessed in drug efficacy studies (6 mice for each treatment group). Mice were then randomly assigned to the treatment groups and treated by an intravenous injection (IV) of 2 mg/kg cisplatin every seven days at 3 doses (Q7D × 3), an intraperitoneal injection (IP) of 150 mg/kg gemcitabine every seven days at 4 doses (Q7D × 4), or the combination thereof. Mice were monitored for tumor growth and alterations in weight (clinical parameters). Tumor size was determined with a caliper, and tumor volumes were calculated using an ellipsoidal formula (1/2 × length × width squared). The data were plotted using Prism software version 7.0 (GraphPad Software) and refer to the mean ± SEM of tumor volume ratio of six mice per group (*n* = 6). The mice were sacrificed when the tumor length reached 20 mm in any direction or achieved a weight loss of over 20%.

In Vitro drug treatment: nine PDX tumor spheroids from a 6-day culture seeded with 1000 cells/well were treated with the antineoplastic drugs cisplatin, gemcitabine, and their combination thereof at a concentration of 10 μM (0 h). Negative and positive control groups were cultured with RPMI culture medium only or supplemented with 20% of dimethyl sulfoxide (DMSO). After 24, 48, and 72 h of treatment, the cell viability of three spheroids (for each treatment group and the control) were assessed using the CellTiter-Glo 3D Assay (Promega, Madison, Wisconsin, USA), following the manufacturer’s protocols. Viability was calculated according to the equation: $\left(\frac{S-PC}{NC-PC}\right)\times 100\%$, where *S*, *PC*, and *NC* are the sample, positive control, and negative control luminescence records, respectively. The experiment was repeated twice. No media change was performed throughout the experiment. The drug concentration was chosen based on previous group work with bladder tumor spheroids [13]. The results of cell viability were plotted using the software for data analysis Prism version 7.0 (GraphPad Software) and displayed as mean ± SEM, referring to two experiments with three technical replicates (wells) each per condition (*n* = 6).

### 3.5. Statistical Analysis

For statistical analysis of the in vitro drug response, the data were analyzed by Prism software version 7.0 (GraphPad Software), using Student’s *t*-test (*p* < 0.01) for independent samples.

## 4. Conclusions

Recent researches have shown that tumor spheroids may be able to recreate the pathophysiological conditions present in vivo in solid tumors and can be considered a good in vitro model for drug response assays. The results obtained in this preliminary work revealed that regular and round-shaped 3D tumor spheroids were generated from in vivo PDX BL0293 and BL0808 models. Moreover, when PDX tumor spheroids were treated with anti-neoplastic drugs, we observed a response profile similar to those obtained in vivo using the PDX models from which the spheroids were derived. Therefore, the in vitro 3D model using PDX tumor spheroids can be a valuable tool to predict the outcome of in vivo drug screening assays and represents a low-cost strategy for the preliminary identification of the optimal treatment for individual patients.
